# Technique of the transcervical-subxiphoid-videothoracoscopic maximal thymectomy

**DOI:** 10.4103/0972-9941.38911

**Published:** 2007

**Authors:** Marcin Zieliński, Łukasz Hauer, Jarosław Kużdżał, Witold Sośnicki, Maria Harazda, Juliusz Pankowski, Tomasz Nabiałek, Artur Szlubowski

**Affiliations:** Department of Thoracic Surgery, Zakopane, Poland; 1 Department of Pathology, Zakopane, Poland; 2 Department of Anesthesiology and Intensive Care Medicine of Pulmonary Hospital, Zakopane, Poland

**Keywords:** Mediastinum, myasthenia, thymectomy, thymus, video-assisted thoracoscopic surgery, videothoracoscopy

## Abstract

**Background:**

The aim of this study is to present the new technique of transcervical-subxiphoid-videothoracoscopic “maximal”thymectomy introduced by the authors of this study for myasthenia gravis.

**Materials and Methods:**

Two hundred and sixteen patients with Osserman scores ranging from I–III were operated on from 1/9/2000 to 31/12/2006 for this study. The operation was performed through four incisions: a transverse 5–8 cm incision in the neck, a 4–6 cm subxiphoid incision and two 1 cm incisions for videothoracoscopic (VTS) ports. The cervical part of the procedure was performed with an open technique while the intrathoracic part was performed using a video assisted thoracoscopic surgical (VATS) technique. The whole thymus with the surrounding fatty tissue containing possible ectopic foci of the thymic tissue was removed. Such an operation can be performed by one surgical team (the one team approach) or by two teams working simultaneously (two team approach). The early and late results as well as the incidence and localization of ectopic thymic foci have been presented in this report.

**Results:**

There were 216 patients in this study of which 178 were women and 38 were men. The ages of the patients ranged from 11 to 69 years (mean 29.7 years). The duration of myasthenia was 2–180 months (mean 28.3 months). Osserman scores were in the range of I–III. Almost 27% of the patients were taking steroids or immunosuppressive drugs preoperatively. The mean operative time was 201.5 min (120–330 min) for a one-team approach and it was 146 (95–210 min) for a two-team approach (*P* < 0.05). While there was no postoperative mortality, the postoperative morbidity was 12%. The incidence of ectopic thymic foci was 68.4%. The rates of complete remission after one, two, three, four and five years of follow-up were 26.3, 36.5, 42.9, 46.8 and 50.2%, respectively.

**Conclusion:**

Transcervical-subxiphoid-VTS maximal thymectomy is a complete and highly effective treatment modality for myasthenia gravis. The need for sternotomy is avoided while the completeness of the operation is retained.

## INTRODUCTION

The beneficial effects of thymectomy in myasthenia gravis (MG) are generally recognized by most neurologists and thoracic surgeons although no prospective, randomized study has been performed to compare the results of operative and conservative treatments for the disease. The choice of the technique of thymectomy is still a matter of debate. There are several methods of thymectomy performed through the trans-sternal, trans-cervical, videothoracoscopic (VTS) and subxiphoid approaches.[1–10] In this report, we present the technique of transcervical-subxiphoid-VTS “maximal”thymectomy developed by the authors of this study.[11]

## PATIENT SELECTION

Patients who were operated on in this study had types I–III of myasthenia gravis according to the Osserman Genkins classification (I = ocular signs and symptoms; II and III: mild and moderate generalized weakness respectively). During the period between 1/9/2000 and 31/12/2006, patients with thymoma and those on whom thymectomy was being repeated (rethymectomy), were operated on using a technique of extended trans-sternal thymectomy, similar to the technique described by Bulkley.[6] If the myasthenia was severe and the clinical state of the patient was not stable, preliminary treatment modalities such as steroids (1 mg/kg/day of prednisone), immunosuppressive drugs (Azathioprine), intravenous immunoglobulins or plasmapheresis were used until the patient's clinical state became optimal. Operating time and intraoperative and postoperative complications were recorded.

## SURGICAL TECHNIQUE

The operative technique of the procedure used is as follows: the patient was positioned supine on the operating table with a roll placed beneath the thoracic spine to elevate the chest and to hyperextend the patient's neck. Under general anesthesia, an endobronchial tube was inserted to conduct selective lung ventilation during the latter part of the procedure. To shorten the operative time and to facilitate performance of the procedure, the operation may be performed by two teams-one called the “cervical team” working from above and the second called “the subxiphoid team” working from below the sternum with control of the videothoracoscope (VTS). Alternatively, the whole operation can be performed by one surgical team performing “the cervical” and “the subxiphoid” parts of the operation sequentially. All operative steps have been described here without specifying if one or two teams were involved.

**Figure 1:**
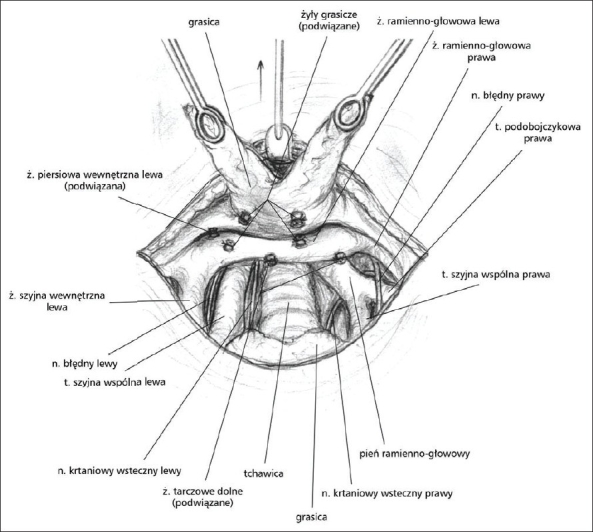
Dissection of the thymus - view of the cervical incision

**Figure 2:**
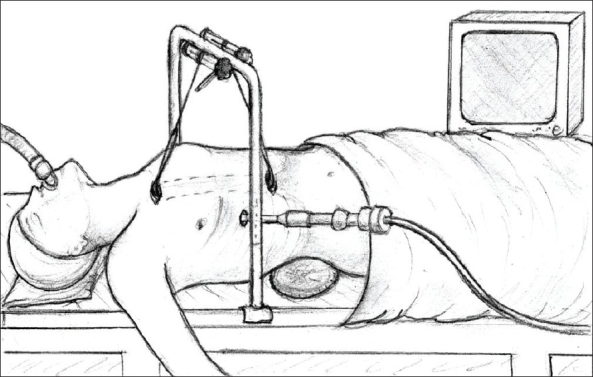
View of the sternal retractor connected to the frame with a traction mechanism to elevate the sternum

**Figure 3:**
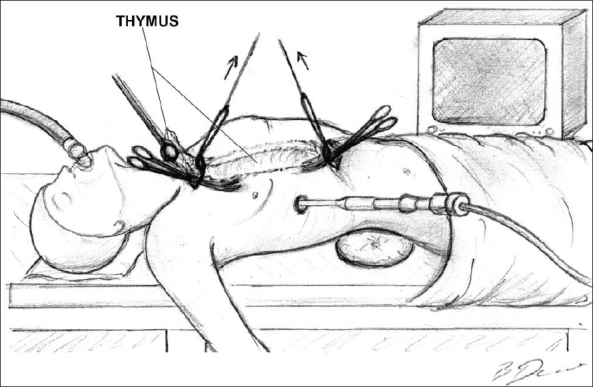
Dissection of the thymus is performed by two teams operating simuultaneously

*Cervical part of the operation:* A transverse 5–8 cm incision was made in the neck above the sternal notch. The platysma and superficial cervical fascia were divided and the anterior jugular veins were also divided and suture-ligated. The strap muscles were split along their median raphe and retracted laterally. The whole thyroid gland was visualized and all foci of the adipose tissue were removed downwards from the level of the upper poles of the thyroid gland. The parathyroid glands and both laryngeal recurrent nerves were visualized and carefully preserved. The fatty tissue containing the superior poles of the thymus was separated from the lower poles of the thyroid gland with 1–4 inferior thyroid veins ligated and divided. Alternatively, devices such as a harmonic knife, LigaSure or vascular clips could also be used to secure the vessels throughout the procedure. The thymus with the surrounding fat was then separated from the sternohyoid and sternothyroid muscles, the trachea, the internal surface of the sternum, the carotid arteries, the innominate artery, the aorta and the right innominate vein [Figure 1]. At this point, a sternal retractor connected to the firm frame with a traction mechanism was inserted under the manubrium of the sternum to elevate it several centimeters to provide access to the anterior mediastinum [Figures 2 and 3]. The lower thyroid veins (1–4) and the thymic veins (1–4) were dissected, clipped and divided close to the left innominate vein. The fatty tissue from the area called “the aorta-caval groove” was removed. The boundaries of this space are the division of the innominate artery and the aorta (medially), the trachea (posteriorly) and the right innominate vein and the right mediastinal pleura (laterally) and the right main bronchus, the azygus vein and the superior vena cava (inferiorly). The dissection proceeded caudally below the left innominate vein and the specimens were separated from the pericardium at a distance of several centimeters. The most difficult but very important part of this operation was the dissection of the adipose tissue from the aorta-pulmonary window. Further dissection of two other branches of the left innominate vein, namely, the left internal thoracic vein and the accessory hemiazygos vein was mandatory. These two veins were subsequently divided and their ends were secured with clips or preferably sutures. The division of these veins provided much better access to the aorta-pulmonary window above the left innominate vein, which was retracted towards the aorta. The next step was the visualization of the left phrenic nerve, which runs very close to the left internal thoracic vein and the left vagus nerve, which runs laterally to the left common carotid artery. With blunt dissection using a peanut sponge, the fatty tissue containing the aorta-pulmonary window was dissected from these nerves, the aorta and the left mediastinal pleura. The left pulmonary artery was visualized at the bottom of the aorta-pulmonary window. In difficult cases, the dissection of the aorta-pulmonary window was completed at a later stage of the operation with a videothoracoscopic camera inserted inside the chest.

*Subxiphoid part of the operation:* A transverse 4–6 cm incision was made above the xiphoid process. The subcutaneous tissue was cut and the medial parts of the rectus muscles were cut near the insertions to the costal arches. The xiphoid process was divided transversely and left without removal. Selective left lung ventilation was started resulting in the collapse of the right lung. The anterior mediastinum was opened from below the sternum. A second sternal retractor connected to the traction frame (the same as one which is used for traction of the manubrium) was placed under the sternum, which was elevated to facilitate access to the anterior mediastinum from below. A port for a 5 mm, 30° oblique thoracoscope was inserted into the right pleural cavity in the 6th intercostal space in the anterior axillary line. The right mediastinal pleura was cut near the sternal surface up to the level of the right internal thoracic vein, which was left intact. The prepericardial fat and right and left epiphrenic fat pads were dissected from the pericardium and diaphragm with blunt dissection using a peanut sponge and a sharp dissection using scissors. Dissection of the prepericardial fat containing the thymus gland proceeded upwards under the control of the VTS camera in an *en bloc* fashion without any attempt to dissect the thymus gland separately. The right phrenic nerve was now at the margin of the dissection. At this moment, the thymus was attached to the pericardium only with its left lower pole. Ventilation of the right lung was resumed and the ventilation of the left lung was disconnected. A port for a 5 mm, 30° oblique thoracoscope was inserted into the left pleural cavity as on the right side. The operating table was then rotated on the right side with an elevation of the left side, which lowered the mediastinum, improving access to the left pleural cavity. Under the control of the VTS camera, the left mediastinal pleura was divided along the sternum and the left prepericardial fat was dissected from the pericardium above the level of the previously divided left internal thoracic vein. The left lower pole of the thymus was separated from the pericardium and a specimen was removed. If necessary, dissection of the aorta-pulmonary window was completed, at this stage of the operation. Hemostasis was checked, the VTS ports were removed and the chest tubes were inserted into both pleural cavities through the incisions made for insertion of the ports. Ventilation of both lungs was resumed and the cervical and subxiphoid incisions were closed in the standard manner. Generally, a patient was extubated immediately after the operation.

## FOLLOW-UP

To estimate the later results of this treatment of myasthenia, questionnaires inquiring into the clinical state and antimyasthenic drug intake were sent to all patients at yearly intervals. Based on the answers to the questionnaires, complete remission rates (lack of myasthenic symptoms with no need for any myasthenic drugs including corticosteroids and other immunosuppressive drugs), improvement rates, no improvement rates, deterioration rates and the late mortality rates (the death from MG or other causes) were calculated.

## RESULTS

**Figure 4:**
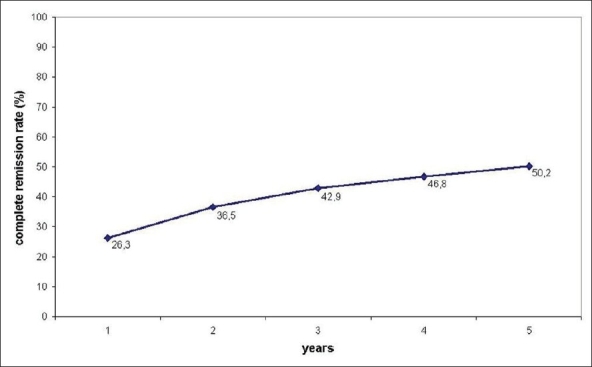
Complete remisssion rates of myasthenic symptoms after one, two, three, four and five years’ follow-up after transcervical-subxiphoid-VATS “maximal” thymectomy

Two hundred and sixteen patients of which 178 were women and 38 were men, all aged 11–69 years (mean 29.7 years), were included in this study. The duration of myasthenia was 2–180 months (mean 28.3 months) and their Osserman scores were in the range of I–III. Almost 27% of the patients were taking steroids or immunosuppressive drugs preoperatively. The mean operative time was 201.5 min (120–330 min) for a one-team approach and 146 (95–210 min) for a two-team approach (*P* < 0.05). While there was no postoperative mortality, the postoperative morbidity was 12% with the incidence of ectopic thymic foci being 68.4%. The rates of complete remission after one, two, three, four and five years of follow-up were 26.3, 36.5, 42.9, 46.8 and 50.2%, respectively [Figure 4]. Table 1 shows the morbidity encountered in the series.

**Table 1:** Complications of transcervical-subxiphoid-VATS maximal thymectomy in 216 myasthenia gravis patients

Type of complications	n (%)
Superior vena cava or left innominate vein laceration (managed with clips or sutures without sternotomy)	2 (0.9)
Postoperative bleeding necessitating revision	5 (2.3)
Temporary laryngeal recurrent nerve paresis	3 (1.4)
Permanent laryngeal recurrent nerve paresis	0
Pleural hematoma necessitating VTS	1 (0.5)
Pleural hematoma necessitating needle aspiration	1 (0.5)
Respiratory insufficiency - ventilator	11 (5.1)
Pneumonia without respiratory insufficiency	1 (0.5)
Minor wound complications	4 (1.9)
Subarachnoid hemorrhage	1 (0.5)
Pneumothorax	1 (0.5)
Overall	30 (13.9)

## CONCLUSIONS

The transcervical-subxiphoid-VTS maximal thymectomy is a highly extensive procedure, performed partly in the open fashion, avoiding the use of sternotomy. A two-team approach helps to shorten the operative time for this procedure.
